# Chronic Stomach Ulcer; Its Surgical Treatment

**Published:** 1908-08

**Authors:** Edward G. Jones

**Affiliations:** Professor of Surgery in the Atlanta School of Medicine


### CHRONIC STOMACH ULCER; ITS SURGICAL TREATMENT.*

BY EDWARD G. JONES, A. B., M. D., PROFESSOR OF SURGERY IN THE ATLANTA SCHOOL OF MEDICINE.

This paper contemplates a discussion solely of:

I. The diagnosis and treatment of chronic gastric and duodenal ulcers;

II. A brief notice of some objections urged against the operative treatment of these ulcers.

No consideration is intended to be accorded such related pathologic conditions as perforation, adhesions, pyloric stricture, etc., although it is acknowledged that these sequelae constitute a natural, and almost necessary, part of any intelligent study of the subject as a whole. Nor will any effort be made to distinguish clinically between gastric and duodenal ulcers

### I.

The symptoms upon which an opinion may be based with reasonable certainty before operation are, in the order of their diagnostic value:

(a) History of intractable dyspepsia.
(b) Hematemesis or melena.
(c) Pain.
(d) Epigastric tenderness.
(e) Regurgitation of sour material.

---

*Read before the Fulton County Medical Society, June 18th, 1907.

(f) Vomiting.

(g) Hyperchlorhydria.

(h) General impairment of health.

(a) Practically every patient who is the subject of a gastric ulcer comes to the doctor with a history of *dyspepsia. Every single case of chronic intractable indigestion should be considered with stomach ulcer in mind.* Every thinking physician can recall cases of years ago which he now knows were stomach ulcer—and some of these patients are dead as a result of the failure to recognize and properly treat the condition.

(b) It is practically inconceivable that a stomach ulcer should have developed without *hemorrhage,* slight or otherwise, at some time in its history. Therefore, hematemesis or melena, or both, are very common symptoms . However, the amount of blood may be so small that its detection is impossible except with the microscope or by chemical tests; and it may, of course, be quite impossible to obtain a specimen in which there is any blood at all. Actual visible hemorrhage occurs in fifty to eighty per cent. of cases.

(c) *Pain* is usually described as burning and gnawing in character. It radiates from the epigastrium as the chief point of intensity. Its appearance some time after the ingestion of a meal rather than immediately is evidence suggesting pyloric or duodenal location of the ulcer.

(d) *Epigastric tenderness* can almost always be elicited upon pressure.

(e) *Gastric regurgitation* is a very common symptom indeed. The patient often accompanies the description of his ills with the statement that he frequently wakes at night to find himself in the act of throwing his head over the side of the bed to spit out a mouthful of sour material. There is hardly any point in the history of the patient which is considered by the Mayos as more significant than this.

(f) *Vomiting* is frequently induced by food, which acts as an irritant to the sensitive ulcer. Indeed, this feature may reach the gravity of marked inanition.

(g) *An excess of HCl* in the vomitus or in a test meal is confirmatory evidence of ulcer.

Of course, no physician would expect to find all of these symptoms exhibited with typical clarity in any one case. Indeed, a minority of cases exhibit all of them in any degree.

Regarding the tripodal relation sustained by the three symptoms of pain, hemorrhage and vomiting to the diagnosis, Mayo Robson says "I have seen and operated on quite a number of cases in which pain had not formed a prominent symptom, in which hematemesis had been entirely absent, and vomiting had not come on until stenosis of the plyorus had produced dilatation."

However, after the clinical diagnosis has been made and operation decided upon, there remains the necessity of confirming the diagnosis by actually recognizing the ulcer with the eye; and a most vital question which confronts every beginner is: How shall I recognize the ulcer, if it be present, without opening the stomach? This ordinarily is not difficult. The esrous coat is adherent to the base of the ulcer and there is, consequently, a characteristic cicatrized, puckered area with indurated margins and clearly evident externally.

Should the ulcer not be found anywhere on the anterior wall of the stomach or in the region of the duodenum, the finger should be thrust through the gastro-hepatic omentum or through the mesentery of the transverse colon and the posterior wall subjected to thorough palpation. Only occasionally does it happen that one is left in doubt about whether or not the ulcer is present. It is conceivable, of course, that the serous coat may not be involved to a point which leaves no doubt. There may be slight suspicious adhesions and a suspicion of induration in the muscular wall. Under such circumstances it is probably better to open the stomach and actually examine its internal surface before proceeding. Only recently the writer felt obliged to do this when the presence of an ulcer was doubtful. All authorities are now agreed that it is unwise to perform gastro-enterostomy unless the ulcer can be actually demonstrated at the time of operation.

Treatment.—There is no longer any doubt as to whether the proper treatment of the average chronic gastric ulcer is medical or surgical. The mortality following the proper surgical treatment has been reduced to about one per cent.; and the number of patients remaining entirely cured after a lapse of two years or more in the clinics of the Mayos, Moynihan and Mayo Robson totals 90 out of every 100; whereas, under medical treatment alone stomach ulcer recurs or relapses so often that some 50 per cent. of patients ultimately succumb to the disease or to one or the other of its complications.

*Until a very few years ago it was taught that gastric ulcer was a medical disease except when uncontrollable hemorrhage or perforation demanded surgical intervention.*

The diagnosis, therefore, being confirmed by observation, some operation on the stomach is called for. The nature of this operation depends largely upon the location of the ulcer and takes cognizance of the tremendous liability to subsequent malignant degeneration. Two propositions may be laid down:

(1) Excision of the ulcer is desirable in order to remove the threat of cancer;

(2) Excision will not usually be practiced because (a) probably the majority of these ulcers are duodenal, and duodenal ulcers seldom become malignant; (b) the excision of an ulcer in the pyloric region means to produce a potential stricture, if not to practically obliterate the hylorus; the alternative being to resect the pyloric end of the stomach—which can always be done safely.

The following procedures are practiced by the men of largest experience:

(1) An ulcer proximal to the pyloric region is to be excised and no gastro-jejunostomy done, if it is certain that the excision will not cause subsequent obstruction.

(2) An extensive pyloric ulcer calls for resection of the pylorus if the surgeon be skilled and the patient's condition will allow—this resection being supplemented by gastro-jejunostomy.

(3) Duodenal ulcers or gastric ulcers not removed for any reason are to be treated by simple gastro-jejunostomy.

This last procedure, in fact, in practice, is the method of treatment of a vast majority of these ulcers. This operation has, especially in the last three years, been followed by most brilliant results. It cures stomach ulcers by affording adequate drainage, which is a desideratum at least temporarily in that it prevents stasis and hyperchlorhydria and discourages gastric grinding—allowing physical and chemical rest to the ulcer.

Undoubtedly the posterior no-loop gastro-jejunostomy is the operation of choice, the first part of the jejunum being anastomosed to the posterior wall of the stomach through a slit in the transverse meso-colon. An object always to be kept in mind is the prevention of subsequent regurgitation of bile, and with the technic as perfected chiefly by the Mayos and By Moynihan this sequel practically never supervenes. The Mayos make the stomach incision oblique from above downward and from right to left upon the assumption that the "natural course" of the jejunum is downward and to the left from its point of origin. Moynihan makes a vertical incision in the stomach wall upon the assumption that the "natural course" of the jejunum at its origin is directly downward. In no case should there be an attempt to reverse the jejunum as it is applied to the stomach.

II.

Internists have been quick to condemn the operation of gastro-enterostomy, maintaining that it is too violent an insult to the physiology of digestion. The clinical answer to such a statement is a sufficient answer. I take it that if the patient gains weight and strength, loses his epigastric tenderness, forgets his previous constant indigestion—even forgets that he has a stomach—and leads a comfortable existence with no evidence of impaired metabolism, he may well exercise little concern as to whether his digestion is interfered with or not.

It is claimed, first, that the secretion of the stomach is interfered with. There can be no doubt that gastric acidity is lowered by the operation, but when we remember that hyperacidity is at least one of the causes for the persistence of the ulcer, this decreased acidity subsequent to operation cannot be regretted.

It is objected, in the second place, that the motility of the stomach is impaired. It is probable that when stasis had previously existed the stomach empties itself more quickly after operation than before, but this relief of stasis is certainly something to be desired, whether the food pass through the pylorus or the stoma.

It is urged again that following gastro-enterostomy there is a pathologic presence of bile and pancreatic juice in the stomach. Since these are alkaline secretions it is not entirely unreasonable, as suggested by Patterson, that their presence there is beneficial, at least temporarily, in overcoming the hyperacidity. In those cases, however, where careful technic has been employed it seems hardly probable that bile is habitually present in the fasting stomach.

It is still further objected that the entrance of gastric contents directly into the jejunum through the anastomotic opening lesens the pancheatic flow, and consequently interferes with fat and proteid digestion, since the presence of acid chyme in the duodenum has long been reverenced as the natural stimulus to pancreatic activity. However, since the jejunum and the third portion of the duodenum are developed together from the midgut, it is not surprising that the pancreatic flow is stimulated by gastric contents in the jejunum as well as in the duodenum.

I am fortunately able to present before you a patient on whom some experiments have been made under my direction since I operated on him six months ago. He is shown not because there is anything unusual about his case, but rather because he exhibited prior to the operation every one of the symptoms which have been discussed, because of his exceedingly satisfactory recovery and because of the observations made subsequently. He suffered with persistent dyspepsia for three years. His vomiting for two or three weeks before operation was just as regular as the ingestion of a meal and his pain was particularly gnawing in character. Since the operation was done in January he has been entirely relieved of every one of his unpleasant symptoms and has not vomited a single time even from the ether at time of operation, excepting on one occasion about two months ago when he suffered for a day or two with an attack of acute gastritis from the eating of over-ripe fish. He is not conscious of a digestive apparatus and has gained, according to his own statement, 50 pounds.

Some experiments made upon this patient in reference to the objections previously discussed resulted as follows:

Several observations beginning two months after operation showed an average reduction of total acidity of 16% below the mean normal.

The stomach has been found empty twice five or six hours after meals. Examination three hours after a full meal of meat showed 60 c. c. of gastric contents.

Bile has appeared to be present once as a faint trace and absent three times in tests made from one to three hours after meals.

Examination of his feces showed no apparent increase in fat three times and an increase one time. There is no indication of interference with normal digestion of proteid material.

Therefore, at least so far as this particular patient is concerned, if we may be allowed to judge from his general appearance, from his own subjective symptoms, and from the observations above mentioned, there can be no valid objection whatever to the treatment to which he was finally subjected, while medical treatment practiced for three years had failed. Of course, the clinical and laboratory results in a single case like this cannot be taken as proving anything, yet it is to be remembered that they are quite in accord with the clinical results obtained in great numbers of cases.

### DISCUSSION.

*Dr. W. B. Jones.*—It seems a rather strange thing that by far the greatest amount of surgical work upon ulcer of the stomach has been done by the Mayos in the West. I cannot think that gastric ulcer is less common in this part of the country than there; nor can I think that the average surgeon, under whose observation such patients come, is deficient or thoughtless in his diagnosis. The fact nevertheless remains that comparatively few surgeons in the United States, except the Mayos, have any very large experience in treating gastric ulcer by operation, which is undoubtedly the proper treatment. In this particular section, I do not believe that the average person with a stomach ulcer ever goes to the surgeon or is ever sent there.

While I should not like to put myself in the light of assuming a critical attitude toward the stomach specialists, I fully believe that the vast majority of patients suffering from stomach ulcer go to the stomach specialist and remain under his treatment exclusively. I believe this is the reason there are so few opportunities offered for surgical treatment.

*Dr. J. C. Olmsted.*—In expressing my interest in the subject under discussion, and my appreciations of the treatment accorded it by Dr. Jones, I am led to reflect upon the responsibility of the general practitioner in sending his patients to the proper place for treatment of their stomach troubles if he does not wish to treat them himself. I mean to say that the average practitioner has too little to do with where his patient goes for stomach trouble;

I mean that he frequently fails to exercise the proper care in determining himself what is the matter with his patient's stomach, and therefore fails to exercise his right and his duty toward his patient in that he sends him at once to the stomach specialist when he perhaps needs other treatment than that usually practiced in the office of these most estimable and necessary gentlemen. Far be it from me to reflect in any sense upon their work. There is a very large class of individuals whom they do much good; nevertheless there is also a very large class whose chronic attendance upon the stomach doctor's office makes it remind one of a barber shop.

*Dr. Jones closing*: I had in mind myself the fact that under present conditions everybody with stomach trouble of any kind goes at once to the stomach specialist, who undoubtedly in the majority of instances benefits the patient—and to a degree not done by medicine until the stomach specialist appeared—but it is admittedly a fact that an exceedingly large percentage of patients who are improved under proper diet, rest, etc., have recurrences or relapses; so that they cannot fail to discourage the doctor. If, now, surgical intervention has reached such a point of development that it is without any marked degree of danger, and at the same time is almost certainly curative then, beyond any doubt, these are surgical cases.

The danger following an ordinary gastro-enterostomy is by no means as great as the danger of perforation, hemorrhage, stricture, etc., to say nothing of a life made more or less miserable by the chronicity of the curse. As previously stated, some 90 per cent. of patients subjected to gastro-enterostomy report themselves as entirely free of all previous unpleasant symptoms two years and more after operation; and it is to be remembered that this report is from patients upon whom the operation was done before it reached its present stage of perfection, the inference being that two years hence a like report will be still more encouraging.

---

The Academy of Sciences at Rome, Italy, announces the endowment of a prize of $2,000 to be awarded perpetually every two years for the best work on pure and applied chemistry with special regard to physical and general chemistry. The prize is open to the world.

